# Properties of Two Novel Esterases Identified from Culture Supernatant of *Penicillium purpurogenum* Grown on Sugar Beet Pulp

**Published:** 2016-12-12

**Authors:** Gabriela Oleas, Eduardo Callegari, Romina Sepulveda, Jaime Eyzaguirre

**Affiliations:** 1 Facultad de Ciencias Biologicas, Universidad Andres Bello, Santiago, Chile; 2 BRIN-USDSSOM Proteomics Facility, University of South Dakota, Vermillion, SD, USA

**Keywords:** Esterases, *Penicillium purpurogenum*, Heterologous expression, *Pichia pastoris*, Sugar beet pulp

## Abstract

**Background:**

The filamentous fungus *Penicillium purpurogenum* grows on a variety of natural carbon sources, such as sugar beet pulp, and secretes to the medium a large number of enzymes that degrade the carbohydrate components of lignocellulose. Sugar beet pulp is rich in pectin, and the purpose of this work is to identify novel esterases produced by the fungus, which may participate in pectin degradation.

**Methods and findings:**

Partially purified culture supernatants of the fungus grown on sugar beet pulp were subjected to mass spectrometry analysis. Peptides thus identified, which may be part of potential esterases were probed against the proteins deduced from the fungal genome sequence. The cDNAs of two putative esterases identified were expressed in *Pichia pastoris* and their properties studied. One of these enzymes, named FAET, is a feruloyl esterase, while the other, PE, is classified as a pectin methyl esterase.

**Conclusions:**

These findings add to our knowledge of the enzymology of pectin degradation by *Penicillium purpurogenum,* and define properties of two novel esterases acting on de-esterification of pectin. Their availability may be useful as tools for the study of pectin structure and degradation.

## Introduction

Pectin is one of the polysaccharides present in lignocellulose, and it is found mainly in the primary cell wall of plants [1]. Several components can be identified in pectin structure: 1) Homogalacturonan (a polymer of D-galacturonic acid residues bound by α-1, 4 linkages, which may be methylated and acetylated). 2) RG I (formed by a backbone of alternating α-D-galacturonic acids and L-rhamnoses, linked to arabinan and galactan side-chains and showing acetyl and methyl esterification), and 3) rhamnogalacturonan II (formed by short chains of galacturonic acid residues substituted by complex side-chains of a variety of monosaccharide’s) [1]. The biodegradation of pectin is a complex process, in which a number of different glycanases, esterases and lyases participate [2].

In our laboratory, we have used for a number of years the filamentous fungus *Penicillium purpurogenum* as a model for the study of the enzymology of lignocellulose biodegradation. This fungus grows on different lignocellulose-containing substances, and secretes to the medium a large number of enzymes, which degrade the lignocellulose polysaccharides [3,4]. Among the carbon sources used is sugar beet pulp. This product is composed of 20% cellulose and 50% pectin [5]. In this work, we have aimed at the finding of potential esterases, which may participate in pectin degradation. As a result, two esterases named FAET and PE have been identified, heterologously expressed in *Pichia pastoris* and characterized.

## Methods

### Microbial strains utilized

*P. purpurogenum* strain ATTC MYA-38 was utilized as the source of enzymes. For cloning purposes, *E. coli* DH5 α or TOP10F were used, and heterologous expression was performed in *Pichia pastoris* GS115, supplied in the Easy Select Pichia Expression Kit (Invitrogen, CA, USA).

### Liquid cultures of *P. purpurogenum*

*P. purpurogenum* was grown on Mandels medium as described previously [6] using sugar beet pulp or glucose at 1% as carbon sources. Liquid cultures on sugar beet pulp (12 L) were incubated for 7 days at 28°C in a shaker at 200 RPM. After filtration on cheesecloth and centrifugation, the clear supernatant was concentrated using a Minitan (Millipore, U.S.A) ultrafiltration apparatus (10 KDa cutoff membrane) and Amicon (Millipore) ultrafiltration Centricons, to a final volume of about 20 mL.

### Partial purification of esterases

In order to obtain a crude separation of esterases, the concentrated supernatant was subjected to chromatography. A pseudo-affinity chromatography was performed using Cibacron blue Sepharose: 50 mL of resin were equilibrated in 50 mM citrate buffer pH 4 and 15 mL of the concentrated supernatant were added. The column was washed with 3 volumes of the buffer and then 3 volumes of a gradient (0 to 1 M NaCl) in the buffer were used to elute proteins.

### Zymograms

Polyacrylamide gels were prepared according to Laemmli [7]. Samples (concentrated chromatography column eluates) contained 25 to 100 μg protein in 60 mM Tris HCl buffer pH 6.8, 25% glycerol and 0.1% Bromophenol Blue. The samples were loaded directly on the gel and the electrophoresis was performed at 4°C for 3 hours at 80 V. The gels were then washed 3 times with 50 mM acetate buffer pH 5 and were then placed in 100 mL of the same buffer containing 1 mg MUA (dissolved in absolute ethanol). The appearance of fluorescence bands was photographed under UV light. The gels were then washed with buffer and stained with Coomassie Brilliant Blue. The bands showing enzyme activity were cut and subjected to mass spectrometry analysis.

### Mass spectrometry and bioinformatics analysis

Mass spectrometry was performed by tandem LC/MS/MS as described by Dong et al. [8]. The resulting data were analyzed by MASCOT v. 2.4 using a protein database derived from the *P. purpurogenum* genome (unpublished). Peptides showing a score >18 were considered. The amino acid sequence of each protein found was analyzed by CDD SEARCH (http://www.ncbi.nlm.nih.gov/Structure/cdd/cdd.shtml) in order to establish which proteins contained domains present in esterase families. In addition, each protein identified as potential esterase was subjected to BLASTP (http://blast.ncbi.nlm.nih.gov/Blast.cgi?PAGE=Proteins) to search for homologies with other possible esterases. Alignment with homologous sequences was performed using CLUSTAL Omega (http://www.ebi.ac.uk/Tools/msa/clustalo/). Gene and cDNA sequences were identified using the program Augustus (http://bioinf.uni-greifswald.de/augustus/). Molecular weights and isoelectric points were estimated with EXPASY (https://www.expasy.org/); the presence of a signal peptide was determined with SignalP (http://www.cbs.dtu.dk/services/SignalP/). dbCAN (http://csbl.bmb.uga.edu/dbCAN/) was used to assign the protein sequences found to the CAZY database families (http://www.cazy.org/). Presence of O- and N-glycosylation was analyzed using NetOGlic and NetNglic (http://www.cbs.dtu.dk/services/NetOGlyc/ and http://www.cbs.dtu.dk/services/NetNGlyc/), respectively.

### DNA preparation

*P. purpurogenum* was grown on 100 mL of 1% glucose for 5 days. The mycelium obtained was separated by centrifugation, frozen in liquid nitrogen and pulverized with a mortar and pestle. DNA was extracted by means of the Genomic DNA Purification kit (Fermentas). The manufacturer’s instructions were followed in all procedures using kits.

### PCR and overlap extension PCR

PCR was performed with either Taq polymerase (Thermo Scientific) for routine assays and High Fidelity PCR Enzyme mix (Thermo Scientific) for cloning and sequencing. The conditions were as follows: initial denaturation at 95°C for 5 min and 30 cycles of denaturation at 95°C for 30 sec, annealing at 59°C for 30 sec, extension at 72°C for 1 min, and a final extension at 72°C for 10 min. The PCR products were gel purified.

Introns were eliminated using “overlap extension PCR” as described by Topakas et al. [9]. The PCR conditions used were the same as those indicated above. Table 1 lists the primers used for amplification of whole cDNAs and Table 2 those used for overlap extension PCR.

### Cloning and expression in *Pichia pastoris*

Cloning of the PCR products was performed in pGEM-T-Easy (Promega). Competent *E. coli* DH5 α cell were transformed with the resulting plasmids. Transformed cells were plated in LB agar supplemented with 1 μL 50 mg/mL Ampicillin and 1 μL 20 mg/ mL X-Gal. Plasmids from white colonies were extracted using the Plasmid DNA Mini Kit II (Omega Bio Tek) and used as templates for PCR with primers including restriction enzymes recognition sites (Table 3) for directional cloning. The PCR products obtained were cloned in pPICZB and the resulting plasmids were transformed into competent *E. coli* TOP10F. Transformed cells were grown in LB plates containing 100 μg/mL Zeocin. The presence of inserts in the resulting clones was checked by PCR, and they were sequenced in both strands using the Sanger method (Macrogen Inc., Seoul, Korea). The DNA sequences of the genes coding for the two enzymes have been entered in GenBank under the following numbers: *faet*: KP313782; *pe*: KP313784.

The plasmids of *pe* were linearized by means of *SacI*, while those of *faet*, by *Pme*I, and transformed into *Pichia pastoris* by electroporation. Transformed clones were selected in YPD (Yeast extract Peptone Dextrose medium) agar plates supplemented with 100 μg/mL Zeocin. DNA was extracted from the clones to confirm by PCR the presence of the insert. The positive clones were grown in 20 mL BMGY (1% yeast extract, 2% peptone, 100 mM potassium phosphate, pH 6.0, 1.34% yeast nitrogen base with ammonium sulfate without amino acids, 0.02% biotin and 1% glycerol) for 2 to 3 days (200 RPM, 28°C) to obtain sufficient biomass. The cultures were centrifuged and re-suspended in 20 mL of BMMY medium (glycerol is replaced by methanol) to induce enzyme production, incubating for up to 5 days. Aliquots were removed to assay for enzyme activity. Larger scale enzyme production was performed (500 mL volume) with the clones of interest.

### Pectin extraction from sugar beet pulp

The procedure described by Ralet et al. [10] was used. The extraction was performed with water in order to preserve possible methyl and acetyl esters present in the pectin.

## Enzyme Assays

### Qualitative assay of esterase activity

For this purpose, MUA (a substrate utilized by many esterases) was utilized. One mg of MUA (Sigma) was dissolved in 1 mL ethanol and 9 mL 50 mM acetate buffer pH 5 were added. Assays were performed in micro plate wells, using 200 μL of substrate and 20 to 50 μL of enzyme preparation. The formation of fluorescent methyl umbelliferone was followed under UV light.

### Assays with pNP derivatives

The following substrates were utilized: pNP-acetate, pNP-butyrate, pNP-decanoate, pNP-dodecanoate, pNP-myristate, pNP-palmitate (all from Sigma) and pNP-ferulate (Carbosynth). 1 mL stock solution of each compound (100 mM) was prepared in dimethyl sulfoxide. They were used to prepare dilutions (1 to 10 mM) in 100 mM sodium phosphate buffer pH 5.8. Tests were performed in microplates, using between 1 to 20 μg proteins in a total volume of 200 μL. Reactions were incubated at 28°C for 15 or 30 min and absorbance at 405 nm were measured immediately.

### Qualitative assay for pectin methyl esterase with Ruthenium Red (Merck)

The technique described by Chakiath et al. [11] was employed. Various pectins (all from Sigma) were used as substrates: citrus pectin 3%, 55-70% and 85% methyl esterified and apple pectin 70-75% methyl esterified.Agar plates containing the substrates were added 5-10 μL enzyme and then incubated at 37°C from 30 min to 2 hours. The plates were washed and covered with 3 mL 0.5% Ruthenium Red. After 1 min the plates were washed. The presence of red spots indicates positive activity. Ruthenium red binds preferably to the anionic polygalacturonic acid rather than to the methyl-esterified pectin [11].

### Detection of tannases activity with rhodanine

The assay was performed according to Sharma et al. [12] using methyl gallate (Sigma) as substrate. 480 μL 0.01 M substrate in 50 mM citrate pH 5 were added 20 μg protein from culture supernatants. After 30 min at 30°C, 300 μL rhodanine (0.667 g in 100 mL methanol) were added, and the mixture incubated for 5 min at 30°C before addition of 200 μL 0.5 M KOH. Absorbance at 520 nm was measured after an additional incubation (30° for 5 min).

### Liberation of acetic acid from pectin

Reaction mixtures containing 160 μL 50 mM phosphate buffer pH 7, 20 µL 10% sugar beet pulp pectin and 20 μL enzymes were incubated for 18 h at 28°C. Acetate liberation was detected using the Acetic Acid (K-ACETRM) kit from Megazyme. As positive control, an alkaline hydrolysate of the pectin was used; the method proposed by Juturu et al. [13] for acetylated xylan was followed.

### Quantitative assay for pectin methyl esterase activity

The procedure described by Grsic-Rausch and Rausch [14-19] was followed, using 50 mM phosphate buffer pH 7.4. Increase of absorbance (NADH formation) was followed at 340 nm after 15-30 min incubation.

### Protease activity assay

As substrate, 100 mM azo-casein (Sigma) in 50 mM phosphate buffer pH 6 was used. 250 μL azo-casein were mixed with 150 μL enzyme and incubated for 30 min at 37°C. Reaction was stopped with 1.2 mL 1% trichloroacetic acid. The mixture was centrifuged, the supernatant was added 1.4 mL 1 M NaOH and absorbance was measured at 440 nm.

### Inhibition by PMSF

A 100 mM solution of PMSF was prepared in absolute ethanol. Enzyme activity was measured using either pNP-acetate as substrate or the pectin methyl esterase assay, with a final PMSF concentration of 2 mM.

### Structure modeling

The amino acid sequences of the identified proteins were used to construct homology models using Schrodinger’s Prime suite program (http://www.schrodinger.com/Prime/) after elimination of the signal peptides (detected by SignalP). The templates used are indicated for each enzyme in the Results section.

## Results

### Identification of possible esterases in sugar beet pulp culture supernatant

Culture supernatants were subjected to partial purification by chromatography in Cibacron-Blue-Sepharose. Activity was found (using the qualitative MUA assay) in both the washing and the eluate.

Samples of the active fractions obtained in the chromatography were analyzed for MUA activity using zymograms. The bands showing activity were cut and subjected to mass spectrometry analysis. As a result, the genes of two potential esterases (named FAET and PE) were identified. FAET was found in the washing (Mascot score of 19), and PE in the gradient of the chromatography (Mascot score of 88). Both DNA sequences contain introns which were eliminated by Overlap Extension PCR. The resulting cDNA’s were sequenced and the results coincide with the sequences annotated in the genome.

### Heterologous expression in *Pichia Pastoris*

The cDNAs were linked to pPICZB, the resulting plasmids were linearized and were transformed into *Pichia pastoris*. Two clones of each protein were selected and subjected to enzyme induction by methanol. Activity of the supernatants was assayed with MUA; only the clones of FAET showed activity. When assayed for pectin methyl esterase activity (with methyl esterified pectin using the Ruthenium Red method) only the PE clones were active. No activity for both assays was shown by the negative control (*P. pastoris* transformed with the empty vector).

## Properties of FAET

**Figure 1:**
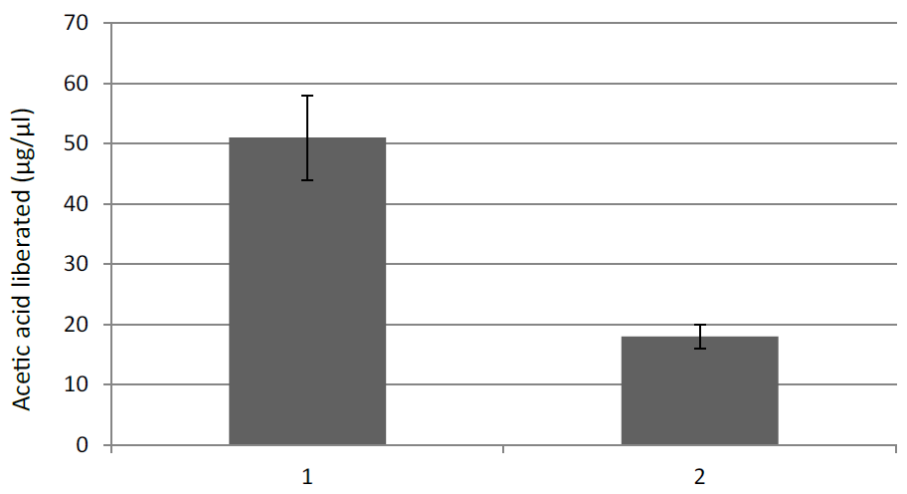
Liberation of acetate from sugar beet pectin by FAET. 20 μl enzymes were incubated with 1% pectin in pH 5.8 phosphate buffers for 16 hours at 28°C. Alkaline hydrolysis was performed in 2N NaOH. 1: alkaline hydrolysis; 2: action of FAET. Assays were performed in triplicate. Bars show standard deviation.

The theoretical molecular mass of FAET was estimated at 56.381 kDa and the pI was estimated at 5.2. The protein presented a signal peptide and five N-glycosylation sites and 3 O-glycosylation sites were identified. The enzyme was active on MUA, pNP-acetate (K_M_=2.3 mM) and pNP-ferulate (K_M_=1.4 mM). The assay with pNP-ferulate was used for optimum pH and the assay with pNP-acetate for optimal temperature determinations. The pH was tested in the range of pH 2.0 to 12.0 (using citrate, Tris-HCl and carbonate buffers), and the effect of temperature in the range from 20°C to 80°C at pH 5.8. The results gave a pH optimum near 8 and an optimal temperature of 38°C. According to the CDD database, FAET belongs to the tannase superfamily. However, FAET did not possess tannase activity when assayed with rhodanine. The enzyme was capable of liberating acetic acid from sugar beet pulp pectin (Figure 1). The control culture of *P. pastoris* lacking the FAET insert showed none of the activities presented by FAET.

## Properties of PE

**Figure 2:**
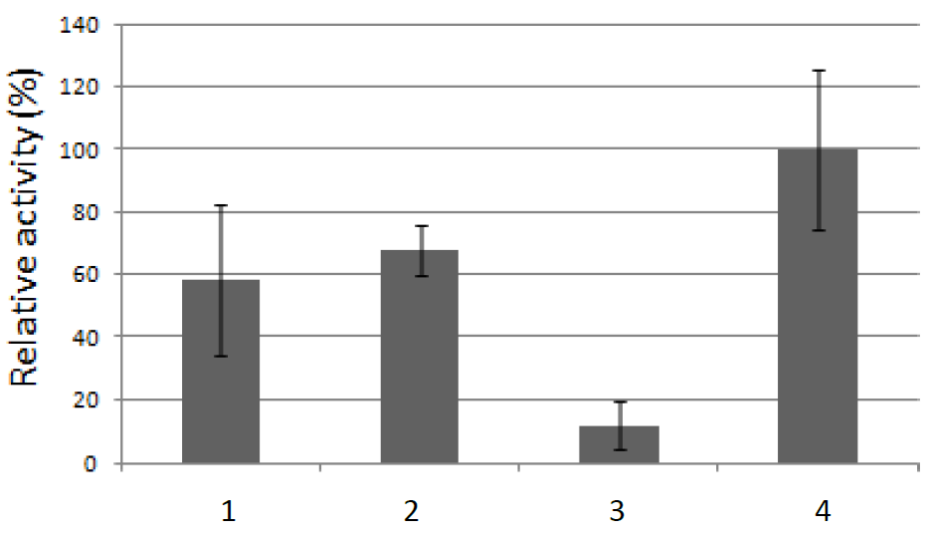
Pectin methyl esterase activity. Assays were performed using the supernatant of a culture of *P. pastoris* transformed with pPICZB-PE. 1: pectin (3% methyl esterified); 2: pectin (55-70% methyl esterified); 3: pectin (85% methyl esterified); 4: sugar beet pulp pectin. Activity was measured with the quantitative assay as described in Methods. Tests were performed in triplicate. Bars show standard deviation.

This enzyme had calculated molecular mass of 32.199 kDa and an estimated pI of 5.4. One N-glycosylation site and 11 O-glycosylation sites were identified, and the enzyme showed a signal peptide. PE presented no activity over MUA and the pNP derivatives of acetate, ferulate, butyrate, myristate, decanoate, laureate and palmitate. It was active in the methyl esterase qualitative test with Ruthenium Red, and using the quantitative assay (see Methods) on citrus pectin with different degrees of methyl esterification and on sugar beet pulp pectin (Figure 2). The quantitative method was used to determine the pH optimum (in the range from pH 3.0 to 9.0) and the temperature optimum (range of 25°-65°C) of PE. Using 3% methyl esterified pectin as substrate, a pH optimum of 5.0 and an optimal temperature of 30°C were obtained for the enzyme. The supernatant of the culture of *P. pastoris* transformed with the plasmid without insert showed none of these activities.

## Discussion

### FAET

**Figure 3:**
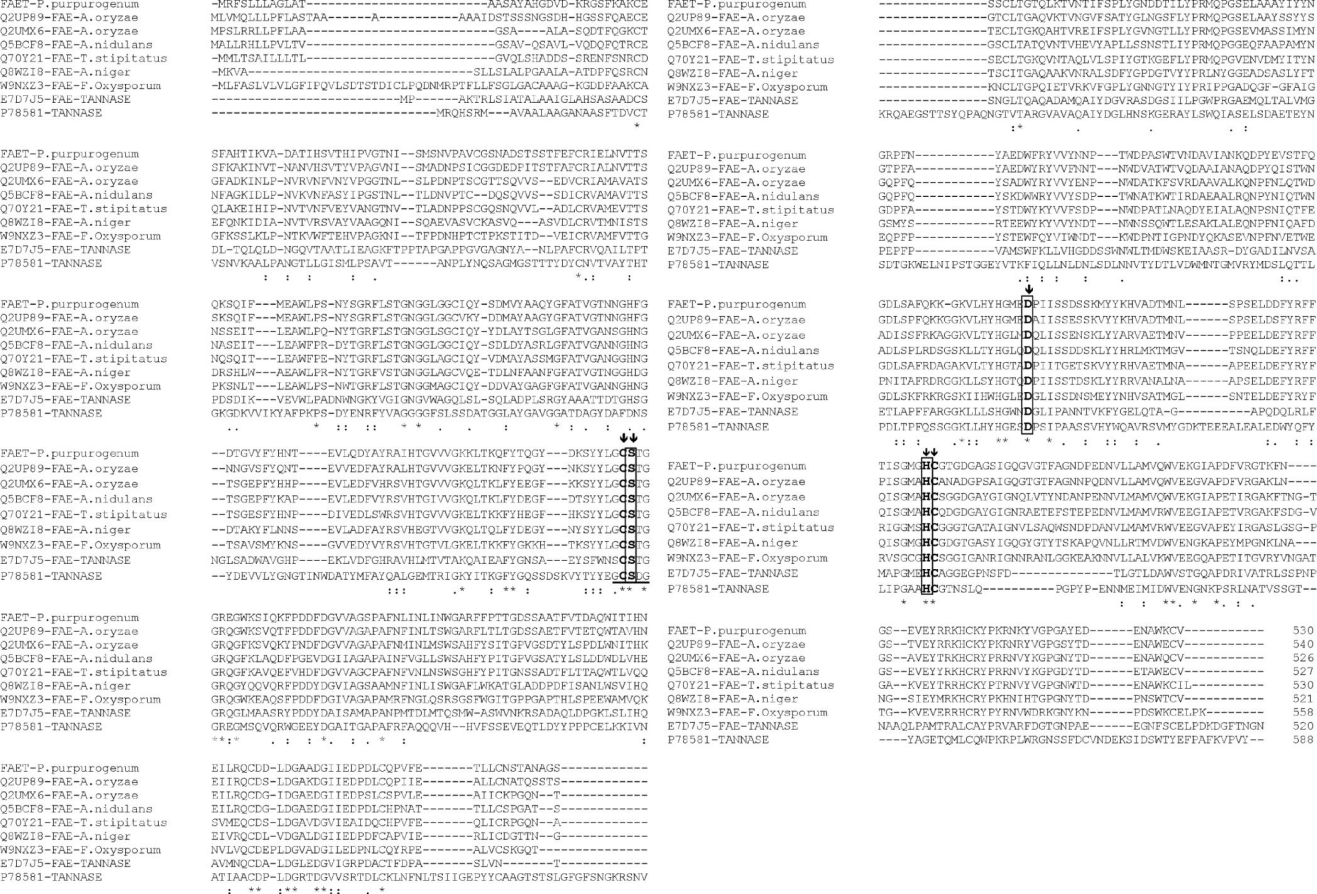
Multiple alignment of the amino acid sequence of FAET with feruloyl esterases and tannases. The catalytic triad is highlighted in rectangles and the nucleophilic elbow is underlined. The residues forming the motif CS-D-HC are marked with vertical arrows. UNIPROT identification numbers for each sequence are shown on the left.

A BLASTP analysis of the amino acid sequence of FAET shows 6 characterized feruloyl esterases with sequence identities ranging from 72% down to 42% (Table 4). All these enzymes belong to the tannase superfamily and possess a tannase domain (Pfam 07519) but lack tannase activity. Only one enzyme is known with both feruloyl esterase and tannase activities; it was isolated from a metagenomic library obtained from a soil sample [20], and it shows only 26% identity with FAET. The sequences of these enzymes are aligned in Figure 3, including, for comparison, a tannase from *Aspergillus oryzae* [21]. The fungal feruloyl esterases and the tannase from *A. oryzae* present the nucleophilic elbow (GXSGX) characteristic of the α/β hydrolases, but it is absent in the enzyme from metagenomic origin. All the sequences possess the motif CS-D-HC, which has been found to be essential for the *A. oryzae* feruloyl esterase activity [22]. dbCAN assigned FAET to CAZy family CE1; however, FAET shows a very low identity with enzymes from this family: only 22% identity is found with a feruloyl esterase from *Aspergillus nidulans* (GenBank: EAA62427). More recently, new classifications of feruloyl esterases have been introduced based on phylogenetic analysis [23,24]; due to its high identity (72%) to the *Aspergillus oryzae* AoFaeB, (UNIPROT Q2UP89), FAET can be classified in family SF1.

**Figure 4:**
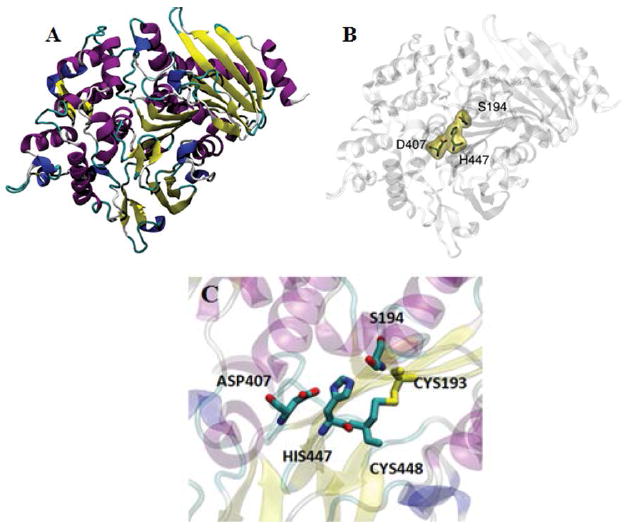
Structural model of FAET. The enzyme from *Aspergillus oryzae* was used as template (PDB 3WMT) A: beta strands are shown in yellow and alpha-helixes in purple. B: the location of the catalytic triad is shown. C: The CS-D-HS motif is highlighted.

*A. oryzae* AoFaeB has been crystallized and its structure determined [22-29]. This structure has been used as a template to build a model for FAET (Figure 4). The structure shows the catalytic triad (S194, D407, H447). That FAET is a serine esterase has been confirmed, since the enzyme is completely inactivated by 2 mM PMSF. The enzyme lacks protease activity, since it is inactive on azo-casein. In short, FAET can be defined as a feruloyl esterase from family SF1.

### PE

By a BLASTP analysis of the sequence of PE, four characterized enzymes were found with identities ranging from 70% to 46%. A comparison of their properties is shown in Table 5. Analysis by dbCAN assigns PE to CAZy family 8, which includes only pectin methyl esterases, in agreement with CDD, which assigns it to the pectin esterase superfamily.

**Figure 5:**
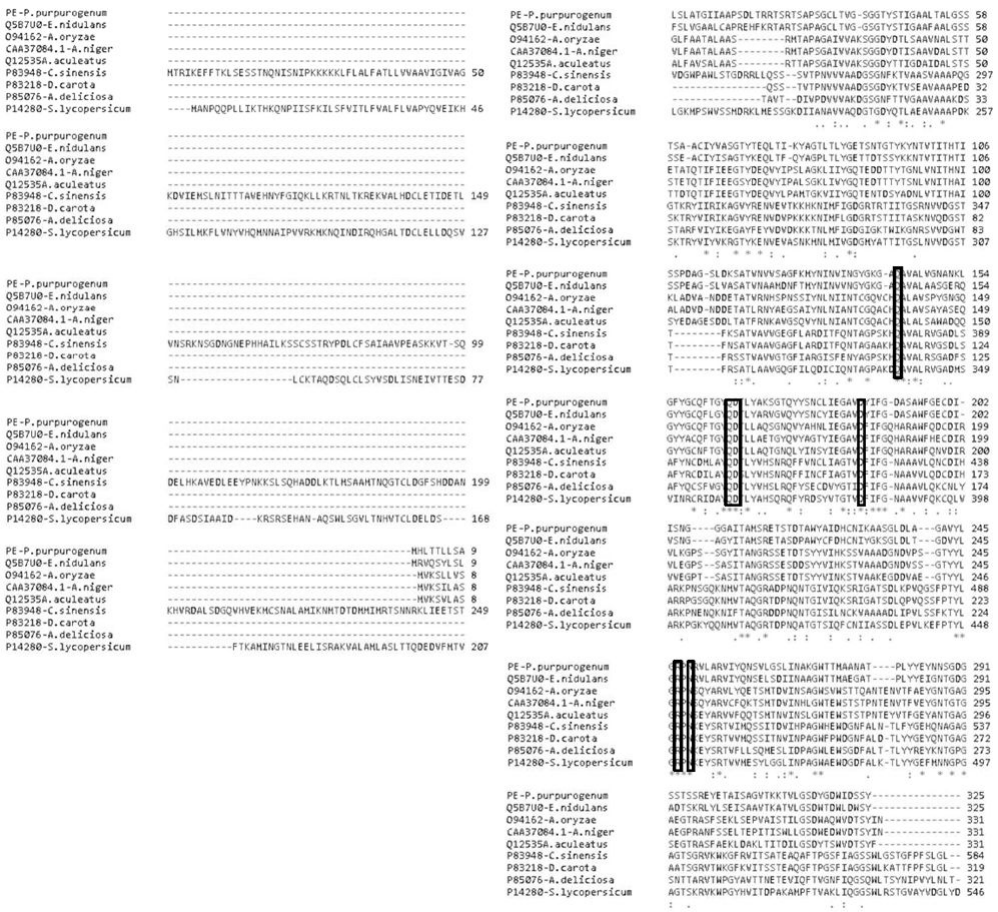
Multiple alignment of the amino acid sequence of PE and other fungal and plant pectin methyl esterases. The active site residues are identified in rectangles. UNIPROT numbers are on the left.

**Figure 6:**
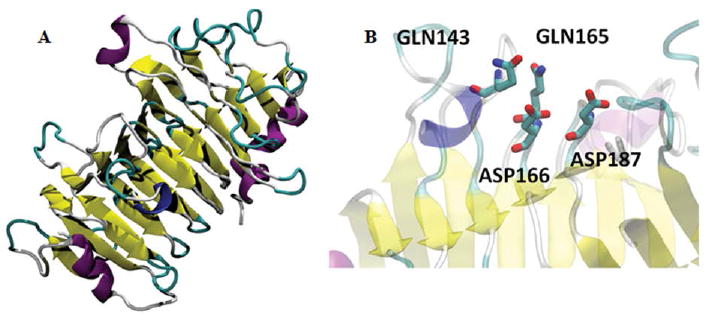
Structural model of PE. The enzyme from *Daucus carota* (PDB 1GQ8) was used as template. A: Model structure. In yellow, the beta-sheets forming the β-helix. B: Active site amino acid residues.

Figure 5 shows the alignment of PE and a set of fungal and plant pectin methyl esterases. Plant sequences are included, since the enzyme from carrot (*Daucus carota*, 31% identity to PE) [29] was used as template to model the structure of PE (Figure 6). The model shows a beta helix structure. Common active site residues were identified, despite of the low sequence identity: an asp residue (D 166) which acts as acid-base, a second asp (D 187) (nucleophile), two gln (Q 143 and 165) forming the anionic hole which stabilizes the transition state, an arg (R 247) and trp (W 249) involved in substrate binding. Both plant and fungi pectin methyl esterases seem to operate by a similar catalytic mechanism, despite different biological functions: plant enzymes participate in fruit ripening [29] while fungal enzymes are involved in the rotting of plant material. PE can thus be identified as a pectin methyl esterase.

In conclusion, two novel esterases have been identified, expressed and characterized. They likely participate in the biodegradation of the pectic component of sugar beet pulp in synergy with other hydrolases and lyases. FAET (feruloyl esterase) may participate in the liberation of ferulic and other cinnamic acid residues present in RG1. PE, a pectin methyl esterase liberates methanol from esters in homogalacturonan and RG1. These enzymes may be valuable tools for a detailed study of pectin degradation.

## Figures and Tables

**Table 1:** Primers used for amplification of esterase cDNAs.

Gene	Primers

*faet*	FAETini: ATGCGGTTTTCGCTG (sense)
	FAETfin: TTAAACACACTTCCAAGCATTC (antisense)

*pe*	PEini: ATGCATTTGACAACGCTCCTTTCAGC (sense)
	PEfin: CTAATAACTCGAATCAATCCAATCACCATA (antisense)

**Table 2:** Primers utilized for “Overlap Extension PCR.”

Gene	Primers

*faet*	FAETini: ATGCGGTTTTCGCTG (sense)
	FAETfin: TTAAACACACTTCCAAGCATTC (antisense)
	FAETOR: ATGTCAGAGTATTGAATACAGCCACCGAGGCCT (antisense)
	FAETOF: CCTCGGTGGCTGTATTCAATACTCTGACATGGTCT (sense)

*pe*	PEini: ATGCATTTGACAACGCTCCTTTCAGC (sense)
	PEfin: CTAATAACTCGAATCAATCCAATCACCATA (antisense)
	PEOF1: AGGGTGCTCAGGCTGTTGCACTCGTCGGCAACGC (sense)
	PEOR1:TATTGGCGTTGCCGACGAGTGCAACAGCCTGAGCA (antisense)
	PEOF2: CAACTGCCTGATCGAAGGAGCCGTGGACTACAT (sense)
	PEOR2: CGAAGATGTAGTCCACGGCTCCTTCGATCAGGCAG (antisense)

**Table 3:** Primers with restriction enzyme recognition sites.

Gene	Sense	Antisense
*faet*	P1.43F: GCGGCCGCATGCGGTTTTCGCTG	P1.43R: GAATGCTTGGAAGTGTGTTTAATCTAGACGC
*pe*	P2.61F: CGAGAATTCATGCATTTGACAACGCTCCTT	P2.61R: GTGATTGGATTGATTCGAGTTATTAGTCTAGACGC

The recognition sequences for restriction enzymes are underlined Primer (sense) for FAET includes a NotI recognition site. Primers (sense) for PE contain an EcoRI recognition site. All antisense primers include a restriction site for XbaI.

**Table 4:** Comparison of the properties of FAET with feruloyl esterases of similar sequence.

Organism (UNIPROT code N°)	References	% Identity with FAET	Optimum pH	Optimal temperature (°C)
FAET	This work	100%	8	38
*Aspergillus oryzae* (Q2UP89)	[15]	72%	6	-
*Aspergillus oryzae* (Q2UMX6)	[15]	57%	6	-
*Aspergillus niger* (Q8WZI8)	[16]	50%	6	-
*Aspergillus nidulans* (Q5BCF8)	[17]	54%	7	45
*Talaromyces stipitatus* (Q70Y21)	[18]	53%	6-7	60
*Fusarium oxysporum* (W9NXZ3)	[19]	42%	6	65

**Table 5:** Comparison of the properties of PE with those of other characterized fungal pectin methyl esterases.

Enzyme source (UNIPROT code N°)	References	% identity with PE	Optimum pH	Optimal temperature (°C)
*Penicillium purpurogenum*	This work	100%	5	30
*Emericella nidulans* (Q5B7U0)	[25]	70%	8	30
*Aspergillus aculeatus* (Q12535)	[26]	44%	4.6	45
*Aspergillus niger* (GENBANK CAA37084.1)	[27]	43%	-	-
*Aspergillus oryzae* (O94162)	[28]	46%	5	55

## Abbreviations

pNP: p-nitrophenyl
DTNB: 5,5′-dithiobis(2-nitrobenzoic acid)
MUA: Methylumbelliferyl acetate
PMSF: Phenyl methyl sulfonyl fluoride
RG I: Rhamnogalacturonan I
